# Clinical and Pathologic Characteristics of Cytologically Indeterminate Thyroid Nodules with Non-V600E *BRAF* Alterations

**DOI:** 10.3390/cancers17050741

**Published:** 2025-02-22

**Authors:** Ryan Instrum, Christina E. Swartzwelder, Ronald A. Ghossein, Bin Xu, Babak Givi, Richard J. Wong, Brian R. Untch, Luc G. T. Morris

**Affiliations:** 1 Head and Neck Service, Department of Surgery, Memorial Sloan Kettering Cancer Center, New York, NY 10065, USA; instrumr@mskcc.org (R.I.); swartzwc@mskcc.org (C.E.S.); givib@mskcc.org (B.G.); wongr@mskcc.org (R.J.W.); untchb@mskcc.org (B.R.U.); 2 Department of Pathology, Memorial Sloan Kettering Cancer Center, New York, NY 10065, USA; ghosseir@mskcc.org (R.A.G.); xub@mskcc.org (B.X.)

**Keywords:** thyroid cancer, molecular testing, BRAF mutation, malignancy risk, clinical outcomes

## Abstract

Background: Molecular assays serve as a potential risk stratification tool for cytologically indeterminate thyroid nodules (ITNs). *BRAF V600E* mutations are nearly always associated with thyroid cancer. However, the malignancy risk for ITNs with other less common *BRAF* alterations is less well understood. In this retrospective cohort study, we examine the risk of malignancy (ROM), histopathologic diagnoses, and clinical outcomes for non-V600E *BRAF*-altered ITNs. Methods: Genomic profiling data obtained from 1034 pre-operative fine-needle aspiration samples from 955 patients were reviewed. Nodules harboring *BRAF V600E* were excluded. Clinical, radiographic, and histopathologic data were analyzed retrospectively from *BRAF*-altered ITNs managed surgically at one comprehensive cancer center (2014–2024). Diagnoses were subdivided based on American Thyroid Association (ATA) risk categories. Results: Thirty-seven patients (3.9%) with non-V600E *BRAF*-altered ITNs were identified (isolated *BRAF* mutation: *n* = 29 [78.4%], *BRAF* + other mutation: *n* = 3 [8.1%], *BRAF* fusion: *n* = 4 [10.8%], *BRAF*-like gene expression: *n* = 1 [2.7%]). All *BRAF* mutations identified in the cohort were class II (*RAS*-independent, intermediate to high kinase activity). Nodules had a median pre-operative diameter of 1.8 cm (interquartile range [IQR] 1.4–2.5). Patients presented with nodal metastases in 2.7% (*n* = 1) of cases, and local invasion was not identified in any patients in the cohort. Approximately half of patients (54.1%) were initially treated with a partial thyroidectomy (lobectomy: *n* = 17 [45.9%], isthmusectomy: *n* = 3 [8.1%]), and the remaining patients underwent total thyroidectomy (*n* = 17 [45.9%]). Median post-operative follow-up was 28 months (IQR 17.8–45.5). ROM for *BRAF* alterations was 73% (95%CI 59–87%; ATA low risk: 64.9%/ATA int risk: 5.4%/ATA high risk: 2.7%). There were no high-risk cancers identified in patients with isolated *BRAF* mutation (benign: *n* = 10 [34.5%], ATA low risk: *n* = 19 [65.5%]), and the most common isolated mutation was *K601E* (*n* = 17, 45.9%) which had a 58.8% ROM (all ATA low risk). Patients with isolated *BRAF* mutations had a significantly lower rate of ATA intermediate or high risk pathology when compared to all other *BRAF* alterations (0% vs. 37.5%, *p* = 0.0072). Only three patients were treated with radioactive iodine post-operatively (8.1%), and no completion thyroidectomy procedures were performed in those who did not initially undergo total thyroidectomy. No patients in the cohort were found to have distant metastatic disease or recurrence, and there were no deaths during the follow-up interval. Conclusions: ITNs harboring non-V600E *BRAF* alterations were rare (3.9% of patients) and typically malignant (73%). Nearly all nodules were benign or ATA low-risk cancers. Only 8% of such nodules were ATA intermediate or high risk cancers. In ITNs with isolated non-V600E *BRAF* and no other genetic alterations, one-third were non-malignant, and all cancers were ATA low risk. In the appropriate clinical context, thyroid lobectomy or active surveillance can be considered for initial management of non-V600E *BRAF*-altered ITNs.

## Simple Summary

Molecular assays are frequently employed as a risk stratification tool for cytologically indeterminate thyroid nodules (ITNs). *BRAF V600E* mutations have been well studied and are nearly always associated with thyroid cancer. However, the prognostic significance of less prevalent *BRAF* alterations is unclear. The aim of this retrospective cohort study is to analyze the clinical and histopathological characteristics of non-V600E *BRAF* alterations to better guide clinical decision-making. Thirty-seven patients with non-V600E *BRAF*-altered ITNs who underwent surgery were identified. Overall, the malignancy rate was 73%, and no patients in the cohort were found to have local invasion, distant metastatic disease, or recurrence after surgery. Among patients with isolated *BRAF* mutation (*n* = 29, 90.6%), 66% of tumors were ATA low-risk cancers, 35% were benign, and none were high-risk cancers. In the appropriate clinical context, thyroid lobectomy or active surveillance can be considered for initial management of non-V600E *BRAF*-altered ITNs.

## 1. Introduction

Thyroid nodules are a common finding worldwide, with an estimated prevalence of 60–70% in the general population [1]. Fine-needle aspiration cytology (FNAC) is the diagnostic reference standard for patients with thyroid nodules. However, FNAC yields indeterminate results (Bethesda III/IV) in 15–25% of thyroid nodules [2,3]. Molecular diagnostics have emerged as a potential risk stratification tool to guide clinical decision-making for cytologically indeterminate thyroid nodules (ITNs) [4,5,6,7,8].

*BRAF* (B-type Raf kinase) gene alterations have been described in many tumor types and are among the most common genetic mutations in thyroid cancer, particularly papillary thyroid carcinoma (PTC). Estimates of *BRAF* mutation prevalence in PTC range from 29 to 83% [9,10,11,12,13]. *BRAF V600E* mutations have been studied extensively, as they alone are detected in 57% of all PTC, making them the most common driver mutation [10]. Moreover, some studies have associated *BRAF V600E* mutations with more aggressive disease and higher cancer-related mortality [14,15].

Despite their low prevalence in ITNs (4.2%), the positive predictive value of *BRAF V600E* mutations is high—close to all *BRAF V600E* mutant thyroid nodules represent PTC [16,17,18]. However, the utility and implications for ITNs harboring non-V600E *BRAF* alterations are less clear, as the literature on these uncommon mutations is limited. Mutations in BRAF are subdivided into three classes based on *RAS* dependency, kinase activity, and dimerization status [19]. Some prior data have suggested that non-V600E mutations in ITNs may be more likely to represent indolent tumors that are follicular-patterned and, in isolation, do not carry the same risk of malignancy or aggressive behavior [20,21,22].

Because of the associations with *BRAF V600E* mutations, most clinicians will generally treat thyroid nodules with less common *BRAF* alterations similarly; however, this may pose a risk of overtreatment if these other nodule genotypes exhibit less aggressive behavior [23]. With more widespread and often reflexive use of molecular diagnostics for ITNs, it is critical to better understand the probability of malignancy and risk profile of ITNs with these alterations. The aim of this study is to analyze the clinical and histopathological characteristics of non-V600E *BRAF* alterations to better guide decision-making.

## 2. Materials and Methods

In this retrospective cohort study, genomic profiling data were reviewed from 955 patients who underwent thyroidectomy at one comprehensive cancer center between January 2014 and January 2024. This study was approved by the Institutional Review Board of Memorial Sloan Kettering Cancer Center.

**Figure 1:**
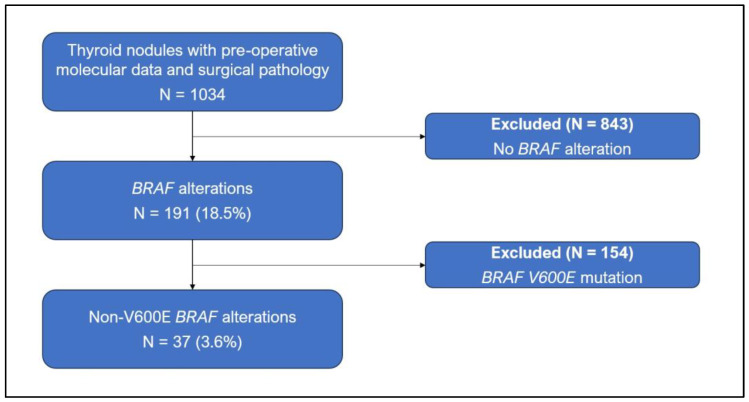
Patient selection process for non-V600E BRAF alteration cohort.

Molecular testing was performed on pre-operative ultrasound-guided FNAC samples from 1034 ITNs. ITNs were classified as Bethesda Category III (Atypia of Undetermined Significance) or Bethesda Category IV [Follicular Neoplasm]) by fellowship-trained cytopathologists. Patients with *BRAF* gene alterations (*n* = 193; total including V600E) were identified using DNA and RNA-based sequencing assays (ThyroSeq v2–v3; CBLPath, Rye Brook, NY, USA). ThyroSeq assays are licensed in the US as laboratory tests that are performed in a CLIA (Clinical Laboratory Improvement Amendments)-approved and CAP (College of American Pathologists)-certified environment. These tests have been validated for the detection of mutations down to 5% allelic fraction (a standard threshold for clinical genomic profiling), and for *BRAF* variants, down to 1% allelic fraction [24]. *BRAF* mutations were categorized into Class I (*RAS*-independent constitutively active monomers), Class II (*RAS*-independent dimers with moderate-to-high kinase activity), or Class III (*RAS*-dependent with low or no kinase activity). Nodules harboring *BRAF V600E* mutations (which would fall into class I) were excluded from this study, leaving 37 patients for analysis (Figure 1).

Surgical pathology reports for the *BRAF*-altered ITNs included in the study were retrospectively examined. All surgical specimens were reviewed by subspecialty head and neck surgical pathologists. Histopathological diagnoses were subsequently stratified into risk categories based on the 2015 American Thyroid Association guidelines. As a quality control measure, findings on histopathology were compared with pre-operative ultrasound, FNAC, and molecular reports by matching nodule size, laterality, and location within the lobe to ensure that the surgical pathology diagnosis rendered corresponded with the nodule that was biopsied and genomically profiled. Incidental malignancies found to be independent from the biopsied nodule were considered separately.

Clinical data from pre-operative, operative, and post-operative timepoints were also retrospectively analyzed for each patient. This included clinical presentation at time of biopsy, operative extent, intraoperative findings, post-operative radioactive iodine (RAI) treatment, and recurrence.

The association between molecular alterations and malignancy was tested using Fisher’s exact test. For all hypothesis testing, significance was set at α < 0.05.

## 3. Results

### 3.1. Patient Characteristics

Overall, 37 patients (3.6% of nodules; 19.2% of *BRAF* alterations) were included in the study (Table 1). The majority of patients were female (*n* = 27, 73.0%), and the median patient age at the time of diagnosis was 44 years (interquartile range [IQR] 36–58). More than half of the nodules were <2 cm (54.1%) on pre-operative ultrasound with a median nodule diameter of 1.8 cm (IQR 1.4–2.5). High-risk sonographic features were identified in two cases (5.4%; irregular margins [*n* = 1], suspicious central compartment lymph nodes [*n* = 1]). One patient (2.7%) presented with both nodal metastasis and gross extrathyroidal extension.

### 3.2. Cytopathologic and Genetic Findings

Cytopathology for the ITNs included in the study was reported as Bethesda III in 62.2% of patients (*n* = 23) and Bethesda IV in the remaining cases (*n* = 14, 37.8%). Genomic profiling revealed non-V600E *BRAF* mutations in 32 nodules (86.5%), 4 *BRAF* fusions (10.8%), and 1 case of “*BRAF*-like gene expression” (2.7%). Among the mutations in the cohort that have been previously characterized, all were class II (*n* = 25, 67.6%), and the remaining mutations are unclassified (*n* = 7, 18.9%). *K601E* was the most common mutation (*n* = 17), representing more than half (53.1%) of all mutations identified. A single *BRAF* mutation was detected in 29 nodules, while the remaining three *BRAF*-mutated nodules (8.1%) harbored co-existing mutations (*K601E* + *EIF1AX p.A113_splice*; *K601N* + *KRAS p.G12D*; *G469* + *TERT p.C228T*).

*BRAF* fusions were detected in four ITNs (10.8%). Three of the identified fusions were *AGK*–*BRAF* (8.1%), and the remaining fusion was *AKAP9*–*BRAF* (2.7%). Molecular testing revealed *BRAF*-like gene expression in one of the included nodules (2.7%).

### 3.3. Histological Diagnoses

**Figure 2:**
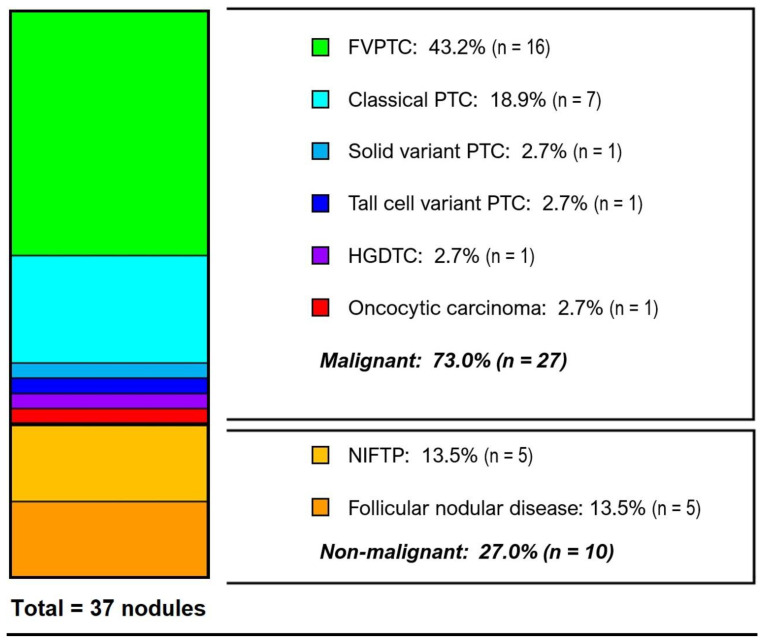
Histological diagnoses of cohort. The overall rate of malignancy (ROM) for BRAF-altered nodules was 73% (95%CI 59–87%, *n* = 27). Papillary carcinoma represented 92.6% of all cancers (*n* = 25, 67.6%). HGDTC, high-grade differentiated thyroid carcinoma; NIFTP, noninvasive follicular thyroid neoplasm with papillary-like nuclear features. Two patients with ATA intermediate-risk and one patient with high-risk DTC were identified in the study cohort (Table 1). One of the intermediate-risk lesions was a high-grade differentiated thyroid carcinoma (HGDTC) with a high mitotic rate associated with co-existing BRAF G469A and TERT mutations. The other was a solid variant of PTC with microscopic invasion of the perithyroidal soft tissues in the nodule with BRAF-like gene expression. The only ATA high-risk lesion in the cohort was a tall cell variant of PTC associated with a AKAP9–BRAF fusion that was found to have extrathyroidal extension (ETE) and lymph node metastases, and this was the only patient with gross ETE or nodal disease in the cohort.

Surgical pathology for the cohort is displayed in Figure 2. The overall rate of malignancy (ROM) for *BRAF*-altered nodules was 73% (95% CI 59–87%, *n* = 27) with the vast majority of cases being histologically low risk by ATA criteria (*n* = 24, 88.9%). Papillary carcinoma represented 92.6% of all cancers (*n* = 25, 67.6%), and the only other histology present was minimally invasive oncocytic carcinoma (*n* = 1, 2.7%) as well as one high-grade differentiated lesion. Follicular (*n* = 16, 43.2%) and classical (*n* = 7, 18.9%) variants (subtypes) of PTC were most common. The histologically benign/non-malignant nodules (*n* = 10, 27.0%) were evenly split between NIFTP and follicular hyperplasia.

In the entire study population, nearly all nodules (*n* = 34, 91.9%) were either non-malignant or ATA low-risk cancers. There were no high-risk cancers identified in patients with isolated *BRAF* mutations (benign: *n* = 10 [34.5%], ATA low risk: *n* = 19 [65.5%]). The most common isolated mutation was *K601E* (*n* = 17, 45.9%) which had a 58.8% ROM (all ATA low risk). Patients with isolated *BRAF* mutations had a significantly lower rate of ATA intermediate or high risk pathology when compared to all other *BRAF* alterations (0% vs. 37.5%, *p* = 0.0072).

Two patients in the cohort underwent central compartment nodal dissection. Via formal and informal nodal sampling, lymph nodes were present in surgical pathology specimens for 19 patients (51.4%). Nodal metastases were only identified in one patient (5.3%) with *AKAP9*–*BRAF* fusion. A total of 53 lymph nodes were analyzed from the other 18 patients, and all were negative for malignancy (ETE) and lymph node metastases, and this was the only patient with gross ETE or nodal disease in the cohort.

### 3.4. Clinical Outcomes

Approximately half of patients (54.1%) were initially treated with a partial thyroidectomy (lobectomy: *n* = 17 [45.9%], isthmusectomy: *n* = 3 [8.1%]), and the remaining patients underwent total thyroidectomy (*n* = 17 [45.9%]). Central compartment neck dissection was performed in only two cases (5.4%). Median postoperative follow-up for the cohort was 27 months (IQR 17.8–45.5). No major operative complications were reported. Only three patients were treated with radioactive iodine post-operatively (8.1%), and no completion thyroidectomy procedures were performed in those who did not initially undergo total thyroidectomy. No patients in the cohort were found to have distant metastatic disease or recurrence, and there were no deaths during the follow-up interval.

## 4. Discussion

*BRAF* mutations are the most common genetic alterations in differentiated thyroid cancer. Among these, *BRAF V600E* is by far the most prevalent, and its clinical implications are well described in the literature [12,14,15,23]. The increased utilization of molecular diagnostics for ITNs has led to the detection of many less common *BRAF* alterations, the prognostic significance of which is not well understood. In this study, we report a series of non-V600E *BRAF* alterations as well as the clinical and histopathologic features that distinguish them from *BRAF V600E*.

Our data indicate that nodules with non-V600E *BRAF* alterations are typically follicular variant PTC (FV-PTC), and the rate of malignancy (73%) is lower than that of *BRAF V600E* (>95%) [16,18]. In particular, the most common non-V600E mutation (*K601E*) had only a 58.8% risk of malignancy and all were ATA low-risk cancers. Overall, the cancers associated with non-V600E *BRAF* were nearly all low risk, particularly in cases of isolated *BRAF* mutations. Isolated *BRAF* mutations, most commonly *K601E*, were either non-malignant (34.5%) or ATA low risk (65.5%), and no isolated non-V600E *BRAF* lesion was found to be intermediate or high risk by ATA criteria. Additionally, no distant metastases, recurrences, or deaths occurred in the cohort. These findings appear to support the limited data published on the subject [20,21,22].

Nodal metastases were identified in only one patient (2.7%) in the cohort with an *AKAP9*–*BRAF* fusion. None of the other patients had clinical or radiographic evidence of nodal disease, and no metastatic disease was found in any of the lymph nodes sampled from these patients (0/53, 0%). These data suggest that those with non-V600E *BRAF* alterations are at low risk for nodal metastasis, and patients are unlikely to benefit from elective neck dissection. This again contrasts the behavior of *BRAF V600E* mutations which have been associated with 2–3x increased risk of lymph node metastasis [25,26].

The one ATA high-risk cancer identified in the cohort was found in a nodule harboring an *AKAP9*–*BRAF* fusion, and histologically this was found to be a tall cell variant PTC with nodal disease and ETE. *AKAP9*–*BRAF* fusions are known oncogenic fusions, often radiation-induced, that leads to constitutively active *BRAF* kinase activity and mitogenic signaling via the MAPK pathway [27,28,29]. Interestingly, the patient with this fusion had no history of radiation exposure or any family history of thyroid disease. This patient underwent total thyroidectomy and central neck dissection with post-operative RAI ablation and had an excellent response with no evidence of residual or recurrent disease. The patient’s most recent surveillance testing showed an undetectable thyroglobulin level with negative thyroglobulin antibodies.

The remaining three fusions in the cohort were *AGK*–*BRAF*, and these were all histologically low-risk PTC (two solid variant, one noninvasive follicular pattern). Additionally, two ATA intermediate-risk cancers were identified. One high-grade differentiated thyroid carcinoma with co-existing *G469A* and *TERT* mutations was found. The other was a solid variant PTC with *BRAF*-like gene expression, and this is likely due to the identification of *V600E*-related gene products.

Important caveats to this study include its limited sample size, its retrospective nature, and single-center design. Although this is the largest series, to our knowledge, of non-V600E *BRAF* mutant thyroid nodules with clinicopathologic correlation, further studies will help to better characterize these nodules, particularly those with less common genotypes (e.g., other than *K601E*). However, in comparison to prior smaller studies, we find that our results are largely congruent: non-V600E *BRAF* alterations are uncommon (1.1–3.6% of ITNs) and typically represent low-risk PTC (70.3–93.1%) that is most often follicular-patterned [20,21,22].

In comparison with *BRAF V600E* mutant thyroid nodules, thyroid nodules with non-V600E *BRAF* mutations have a lower risk of malignancy, and very low risk of aggressive or ATA high-risk malignancy. These data may be helpful in counseling patients and avoiding overtreatment. Given their comparatively indolent nature, isolated non-V600E *BRAF* mutations without other adverse clinical or radiographic findings can be treated first with lobectomy or active surveillance. We note that in those nodules harboring multiple concomitant mutations, that data are limited, and it is unclear if active surveillance is necessarily as appropriate in all of these cases [30,31].

## 5. Conclusions

ITNs harboring non-V600E *BRAF* alterations were usually (73%) malignant, although with lower probability than *V600E* mutations. Nearly all nodules were benign or ATA low-risk cancers. Only 8% of such nodules were ATA intermediate- or high-risk cancers. In ITNs with isolated non-V600E *BRAF* and no other genetic alterations, one-third were benign, and all cancers were ATA low risk. In the appropriate clinical context, thyroid lobectomy or active surveillance can be considered for initial management of non-V600E *BRAF*-altered ITNs.

## Figures and Tables

**Table 1:** Cohort characteristics of 37 patients with non-V600E *BRAF*-altered indeterminate thyroid nodules.

**Age at Diagnosis (Years)**		**Treatment Type**	
Median (IQR)	44 (36–58)	Total thyroidectomy	17 (45.9%)
Range	19–72	Thyroid lobectomy	17 (45.9%)
		Isthmusectomy	3 (8.1%)
**Sex**			
Female	27 (73.0%)	**Final surgical pathology**	
Male	10 (27.0%)	Benign/non-malignant	10 (27.0%)
		Malignant	27 (73.0%)
**Nodule size on US (cm)**		ATA low risk	24 (64.9%)
Median (IQR)	1.8 (1.4–2.5)	ATA intermediate risk	2 (5.4%)
<2	20 (54.1%)	ATA high risk	1 (2.7%)
2–4	16 (43.2%)		
>4	1 (2.7%)	**Histology**	
		Papillary carcinoma	25 (67.6%)
**Bethesda category**		*Follicular subtype*	16 (43.2%)
III	23 (62.2%)	*Classical subtype*	7 (18.9%)
IV	14 (37.8%)	*Solid subtype*	1 (2.7%)
		*Tall cell subtype*	1 (2.7%)
**Alteration type**		NIFTP	5 (13.5%)
*BRAF* mutation	32 (86.5%)	Follicular nodular disease	5 (13.5%)
Class I	0 (0%)	Oncocytic carcinoma	1 (2.7%)
Class II	25 (67.6%)	HGDTC	1 (2.7%)
*K601E*	17 (45.9%)		
*G469A*	4 (10.8%)	**Gross extrathyroidal extension**	1 (2.7%)
*N486_P490del*	1 (2.7%)		
*Class II +* other *	3 (8.1%)	**Nodal disease**	1 (2.7%)
Class III	0 (0%)		
Unclassified ^†^	7 (18.9%)	**Distant metastases**	0 (0%)
*BRAF* fusion	4 (10.8%)		
*AGK*–*BRAF*	3 (8.1%)	**Radioactive iodine**	3 (8.1%)
*AKAP9*–*BRAF*	1 (2.7%)		
*BRAF*-like gene expression ^‡^	1 (2.7%)	**Recurrence**	0 (0%)
**Duration of follow-up (months)**		**Deaths**	0 (0%)
Median (IQR)	27 (17.8–45.5)		

**IQR**, interquartile range; **ATA**, American Thyroid Association; **HGDTC**, high-grade differentiated thyroid carcinoma; **NIFTP**, noninvasive follicular thyroid neoplasm with papillary-like nuclear features; * K601E + EIF1AX (p.A113_splice); K601N + KRAS (p.G12D); G469 + TERT (p.C228T); ^†^ T599del (1); T599_V600insP (1); T599_R603delins? (1); A598_T599insV (1); A598dup (1); T488_Q493delinsK (1); exon 2–8 deletion with fusion of exons 1 and 9 (1); ^‡^ Sample positive for “BRAF-like gene expression alterations associated with thyroid cancer” (ThyroSeq v3 GC).

## Data Availability

Data presented in this study necessary to replicate results are available on request from the corresponding author. Patient-level data are not publicly available due to the ethics approval agreement.
